# Effects of Olympic Combat Sports on Cardiorespiratory Fitness in Non-Athlete Population: A Systematic Review of Randomized Controlled Trials

**DOI:** 10.3390/jcm12237223

**Published:** 2023-11-22

**Authors:** Cristopher Muñoz-Vásquez, Jordan Hernandez-Martinez, Francisco Ramos-Espinoza, Tomas Herrera-Valenzuela, Braulio Henrique Magnani Branco, Eduardo Guzman-Muñoz, Sibila Floriano Landim, Jessica Mondaca-Urrutia, Pablo Valdés-Badilla

**Affiliations:** 1 CESFAM Dr. Juan Carlos Baeza, Departamento de Salud San Clemente, San Clemente 3520000, Chile; cristophermunoz@saludsanclemente.cl; 2 Programa de Magíster en Ciencias de la Actividad Física, Facultad de Ciencias de la Educación, Universidad Católica del Maule, Talca 3530000, Chile; 3 Department of Physical Activity Sciences, Universidad de Los Lagos, Osorno 5290000, Chile; 4 Programa de Investigación en Deporte, Sociedad y Buen Vivir, Universidad de los Lagos, Osorno 5290000, Chile; 5 Programa de Doctorado en Psicología, Facultad de Ciencias de la Salud, Universidad Católica del Maule, Talca 3530000, Chile; ps.franciscoramos@gmail.com; 6 Department of Physical Activity, Sports and Health Sciences, Faculty of Medical Sciences, Universidad de Santiago de Chile (USACH), Santiago 8370003, Chile; tomas.herrera@usach.cl; 7 Postgraduate Program in Health Promotion, Cesumar University, Maringá 87050-390, Paraná, Brazil; braulio.branco@unicesumar.edu.br; 8 School of Kinesiology, Faculty of Health, Universidad Santo Tomás, Talca 3530000, Chile; eguzmanm@santotomas.cl; 9 School of Kinesiology, Faculty of Health Sciences, Universidad Autónoma de Chile, Talca 3530000, Chile; 10 Occupational Therapy School, Faculty of Psychology, Universidad de Talca, Talca 3460000, Chile; sibila.floriano@utalca.cl; 11 Department of Physical Activity Sciences, Faculty of Education Sciences, Universidad Católica del Maule, Talca 3530000, Chile; jmondaca@ucm.cl; 12 Sports Coach Career, School of Education, Universidad Viña del Mar, Viña del Mar 2520000, Chile

**Keywords:** athletic performance, physical fitness, respiratory function tests, combat sports, martial arts, population groups

## Abstract

This systematic review aimed to assess the available body of published peer-reviewed articles related to the effects of Olympic combat sports (OCS) on cardiorespiratory fitness (CRF) in the non-athlete population. The methodological quality and certainty of evidence were evaluated using PRISMA, TESTEX, RoB, and GRADE scales. The protocol was registered in PROSPERO (code: CRD42023391433). From 4133 records, six randomized controlled trials were included, involving 855 non-athletes (mean age = 27.2 years old). The TESTEX scale reported all studies with a ≥ 60% (moderate-high quality) score. The GRADE scale indicated moderate to low certainty of evidence. It was only possible to perform a meta-analysis on direct methods to maximum oxygen consumption (VO_2_max). The main results indicated significant differences in favor of OCS compared to active/passive controls in VO_2_max (SMD = 4.61; 95%CI = 1.46 to 7.76; I^2^ = 99%; *p* = 0.004), while the individual results of the studies reported significant improvements in favor of the OCS on the indirect methods of the CRF. OCS improved CRF in a healthy non-athlete population of different ages, specifically showing a significant improvement in VO_2_max with direct tests, such as cardiopulmonary tests. However, moderate to low certainty of evidence is reported, so no definitive recommendations can be established.

## 1. Introduction 

Cardiorespiratory fitness (CRF) is a powerful marker of general health status in children, youth, adults, and older people [1]. Worldwide, 28% of the population is physically inactive, according to the World Health Organization [2], associated with low levels of CRF, increasing cardiovascular and mortality risk in all age groups [3]. In contrast, regular physical activity has been associated with higher levels of CRF and lower cardiovascular risk, morbidity, and mortality [4,5]. Non-pharmacological therapies such as regular physical activity and/or sports have been shown to lead to high levels of CRF [4,6], with multiple health benefits [7]. For example, primary and secondary prevention of several chronic diseases [8] such as cardiovascular disease [9], diabetes [10], cancer [11], hypertension [12], obesity [13], depression [14], osteoporosis [15], as well as the avoidance of early death [16]. 

In this context, the most common physical activity strategies correspond to walking, cycling, recreational sports, active recreation, dance, and play [2]. While among the least common strategies, we find Olympic combat sports (OCS) such as boxing, fencing, judo, karate, taekwondo, and wrestling, probably because of the stigma of risky activities [17], or else, because they are associated with a greater likelihood of injury in athletes [18]. However, OCS with proper dosage has been reported as an option to improve health status in children [19], adolescents [20], adults [21], and older people [22], being an alternative to traditional physical activity [23], due to the execution of high-intensity intermittent actions with multidirectional movements [24] that predominantly require an aerobic response during the activity [25]. Boxing, judo, karate, and taekwondo have shown positive effects on the physical level, including CRF, through diverse assessments in athletes [26] and non-athlete populations [22,27].

A systematic review with meta-analysis conducted by Linhares, et al. [28] reported significant improvements in favor of combat sports in static (*p* < 0.01) and dynamic balance (*p* < 0.01) compared to control groups in older people. In addition, individual results of the studies with OCS of a systematic review in older people by Valdés-Badilla, et al. [22] reported improved physical measures (i.e., balance, muscle strength, and CRF), without being able to confirm the differences concerning control groups due to the diversity of measurements. Another systematic review by Stamenković, et al. [17] detected significant improvements (*p* ˂ 0.05) in favor of karate, judo, and taekwondo compared to active/inactive control groups in CRF in preschool and school children. Similarly, a randomized controlled trial by Combs, et al. [29] reported significant improvements (*p* = 0.013) in favor of the experimental group that performed an intervention for 12 weeks with three sessions per week by boxing regarding traditional physical activity in the 6-min walk test in adults with Parkinson’s disease. In another study conducted by Brasil, et al. [30], they compared overweight and obese children in a judo intervention for 12 weeks with two sessions per week, registering significant improvements (*p* < 0.05) in maximum oxygen consumption (VO_2_max) in both groups. Also, Cheema, et al. [31] reported significant improvements (*p* = 0.01) in VO_2_max by a 12-week intervention with four sessions per week of boxing compared to a continuous walking intervention in Australian obese adults. On the contrary, in a randomized controlled trial conducted by Roh, et al. [32] in overweight and obese Korean adolescents, no reported significant improvements (*p* = 0.80) in VO_2_max in a 16-week intervention with five sessions per week through taekwondo compared to an inactive control group.

The results were inconclusive due to the variety of instruments used to determine the CRF, such as the characteristics of the population analyzed [29,30,31,32], i.e., the diversity of study design, duration, and dosage of interventions using OCS [17,22], among other factors. A systematic review would allow aggregating the sample size of different studies, providing not only high-quality evidence but also generating new knowledge that can benefit from making more informed decisions to professionals helping to make evidence-based decisions regarding the application of OCS [22]. Similarly, a systematic review can contribute to detecting gaps and limitations in the scientific literature on OCS in the non-athlete population, providing valuable information for researchers. Therefore, this systematic review aimed to analyze the availability of published peer-reviewed articles related to the effects of OCS on CRF in the non-athlete population. The outcomes of this systematic review could be useful for making informed decisions about the impact of this type of practice on the non-athlete population. 

## 2. Methods

### 2.1. Protocol and Registration 

This systematic review followed the Preferred Reporting Items for Systematic Reviews and Meta-analyses (PRISMA) protocols [33]. The protocol was registered in PROSPERO (International Prospective Register of Systematic Reviews; ID code: CRD42023391433).

### 2.2. Eligibility Criteria

The inclusion criteria for this systematic review were original peer-reviewed articles with no language or publication date restriction, published up to September 2023. Excluded records were conference abstracts, books and book chapters, editorials, letters to the editor, protocol records, reviews, case studies, and trials. In addition, the population, intervention, comparator, outcome, and study design (PICOS) framework was followed to incorporate studies into a systematic review (Table 1).

### 2.3. Information and Database Search Process

Seven databases were used for the search procedure, which took place between May 2022 and September 2023: PEDro (Physiotherapy Evidence Database), PubMed, ProQuest, EBSCOhost, CINAHL Complete, Scopus, and Web of Science (core collection). The US National Library of Medicine’s Medical Subject Headings (MeSH) and free language phrases relating to CRF, OCS, and non-athlete populations were used. The following search string was used: (“cardiorespiratory fitness” OR “aerobic fitness” OR “aerobic capacity” OR “cardiovascular health” OR “maximum oxygen consumption” OR “VO_2_max” OR “VO2_max_” OR “VO2max” OR “VO_2_peak” OR “VO2peak” OR “VO2_peak_” OR “cardiorespiratory function” OR “physical fitness” OR “functional capacity” OR “METs”) AND (“boxing” OR “fencing” OR “judo” OR “karate” OR “taekwondo” OR “wrestling” OR “Olympic combat sports”) NOT (“athletes”). The included articles and inclusion and exclusion criteria were sent to two independent experts to help identify additional relevant studies. We established two criteria the experts had to meet: (i) have a Ph.D. in sports science, and (ii) have peer-reviewed publications on CRF in different population groups and/or OCS in journals with an impact factor according to Journal Citation Reports^®^. Experts were not provided with our search strategy to avoid biasing their searches. Once all these steps were completed, we searched the databases on 30 September 2023, to retrieve relevant errata or retractions related to the included studies.

### 2.4. Studies Selection and Data Collection Process

The EndNote reference manager (version X9, Clarivate Analytics, Philadelphia, PA, USA) was used to export the studies. Independent searches were conducted by two authors (CMV, JHM), who also examined titles, abstracts, and complete texts and eliminated duplicates. At this point, there were no disparities discovered. The procedure was carried out once more for reference list searches and recommendations from other specialists. Potentially acceptable articles were then reexamined in full text, and the rationale for eliminating those that did not fit the selection criteria was disclosed.

### 2.5. Methodological Quality Assessment

The methodological quality of the selected studies was assessed with TESTEX [34], explicitly designed for exercise-based intervention studies. TESTEX scores were used as a possible exclusion criterion [34]. It has a 15-point scale (5 points for study quality and 10 points for reporting) [34]. This process was performed independently by two authors (CMV, FRE), and a third author (JHM) acted as a referee for borderline cases, which were then validated by another author (PVB).

### 2.6. Data Synthesis

The following data were obtained and analyzed from the selected studies: (i) author and year of publication, (ii) country of origin, (iii) study design, (iv) initial health of the sample, (v) number of participants in the intervention and control groups, (vi) mean age of the sample, (vii) activities performed in the OCS groups and control groups, (viii) training volume (total duration, weekly frequency, and time per session), (ix) training intensity, (x) CRF data collection instruments, and (xi) the main outcomes of the studies.

### 2.7. Risk of Bias in Individual Studies

The risk of bias (RoB) in individual studies was assessed using the Cochrane risk-of-bias tool for randomized controlled trials (RoB 2) [35]. Two authors (CMV, JHM) independently completed RoB analysis, which was reviewed for another author (PVB). The original articles were re-analyzed, where inconsistencies emerged until a consensus was achieved.

### 2.8. Summary Measures for Meta-Analysis

Meta-analyses were included in the study protocol, with full details available at PROSPERO, registry code CRD42023391433. All analyses were carried out using Review Manager (RevMan 5.4) software to calculate the standardized mean difference (SMD) and the mean difference (MD), a standard statistic that measures the absolute difference between the mean values in two groups in a randomized controlled trial [36]. The SMD and MD of the CRF from preintervention to postintervention, between groups (OCS vs. Control group) in each study [37], were calculated and pooled using the random-effects model (DerSimonian-Laird approach). The underlying assumption of the random-effects model is that samples are drawn from populations with different effect sizes and that true effects differ between studies (interventions and duration). Data were pooled if outcomes were reported by at least three studies [38]. 

### 2.9. Certainty of Evidence

Studies were assessed for certainty of evidence using the Grading of Recommendations, Assessment, Development, and Evaluation (GRADE) scale [39] and classified as having high, moderate, low, or very low certainty of evidence. All analyses started with a high degree of certainty due to the inclusion of studies with randomized controlled trial design and were downgraded if there were concerns about the risk of bias, consistency, accuracy, precision, directness of results, or risk of publication bias [39]. Two authors (CMV, JHM) assessed the studies independently, and any discrepancies were resolved by consensus with a third author (PVB).

## 3. Results

### 3.1. Study Selection

**Figure 1:**
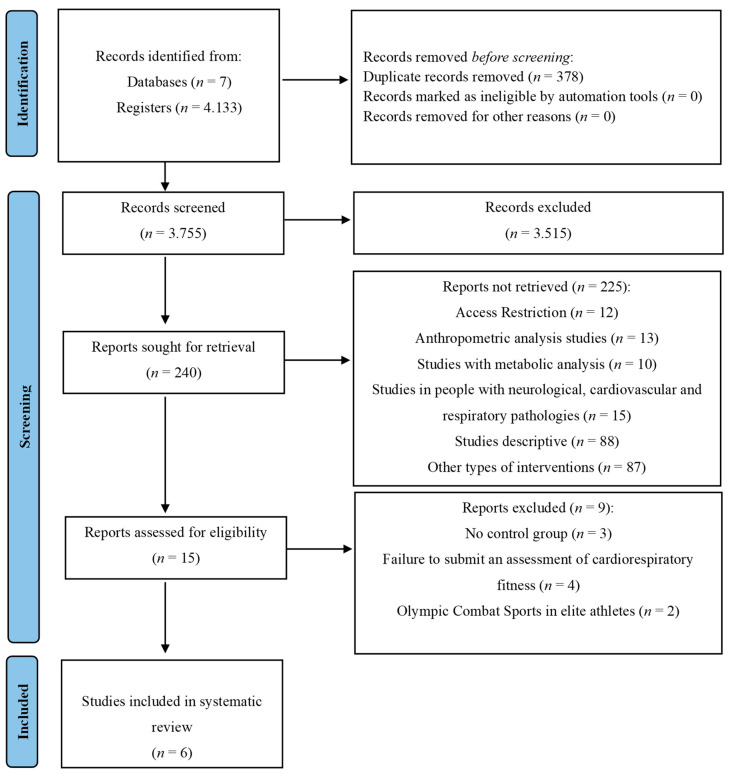
Flowchart of the review process. Legends: Based on the PRISMA-P guidelines [33].

Figure 1 details the search process for the studies. A total of 4133 records were found. Subsequently, duplicates were eliminated, and the studies were filtered by selecting the title, abstract, and keywords, resulting in 3755 references. In the subsequent analysis phase, 3515 articles were excluded because the texts did not meet the search criteria, leaving 240. Subsequently, 225 reports were excluded because they were not retrieved, 12 due to access restriction, 13 studies of anthropometric analysis, 10 studies with metabolic analysis, 15 studies in people with neurological, cardiovascular, and respiratory pathologies, 88 descriptive studies, and 87 studies of other types of interventions. After this process, 15 potential studies remained, of which 3 were excluded for not having a control group, 4 for not presenting CRF assessment, and 2 for OCS in elite athletes. Where only 6 met all the selection criteria [23,30,31,32,40,41,42,43,44]. 

### 3.2. Methodological Quality

The six selected studies were analyzed using the TESTEX scale (Table 2). All studies achieved a score equal to or above 60% on the scale, namely 14/15 [31], 11/15 [40,45,46], 10/15 [44], and 9/15 [47], indicating moderate to high methodological quality, therefore no study was excluded from the systematic review.

### 3.3. Risk of Bias within Studies

**Figure 2:**
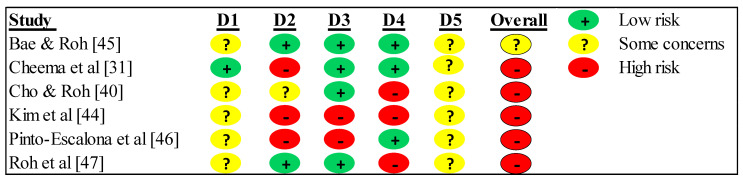
Risk of bias within studies. Legends: D1: randomization process; D2: deviations from the intended interventions; D3: missing outcome data; D4: measurement of the outcome; D5: selection of the reported result.

**Figure 3:**
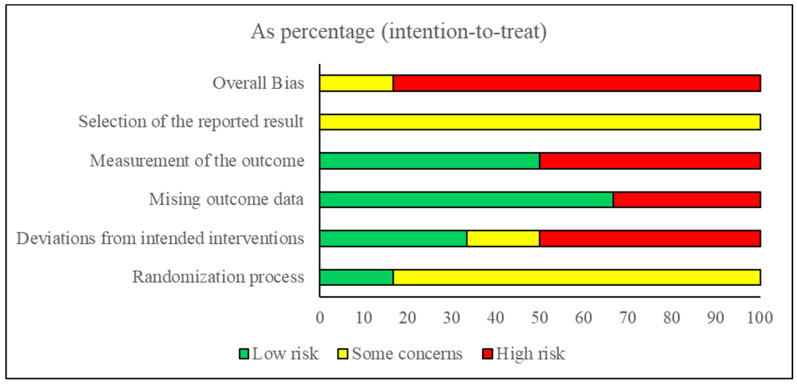
Risk of bias summary: review authors’ judgments about each risk of bias item for each included study.

The risk of bias was high for five studies [31,40,44,46,47]. Only one study showed some concerns [45]. In the randomization process, five studies showed some concerns [40,44,45,46,47], and only one showed a low risk [31]. While in deviations from the intended interventions, two studies showed a low risk [45,47], one study showed some concerns [40], and three studies showed a high risk [31,44,46]. In the missing outcome data, four studies showed a low risk [31,40,45,47], and two showed a high risk [44,46]. In measuring the outcome, three studies showed a low risk [31,45,46], and three showed a high risk [40,44,47]. While selecting the reported outcome, the six studies showed some concerns [31,40,44,45,46,47]. The risk of bias summary is presented in Figure 2, and the risk of bias graph is presented in Figure 3.

### 3.4. Studies Characteristics

The variables analyzed in the six selected studies are listed in Table 3. Three of these studies were conducted in South Korea [40,45,47], one in Australia [31], one in the United States of America [44], and one multicenter study developed with participants from Spain, Portugal, France, Poland, and Germany [46]. All six studies were randomized controlled trials regarding study design [31,40,44,45,46,47]. 

### 3.5. Sample Characteristics

Five studies presented groups of 12 to 37 participants [31,40,44,45,47], while only one study presented 721 participants [46]; the result was a sample of 855 non-athlete population. Composed of schoolchildren aged 7 to 11 years [46,47], high school females and university students aged 15 to 22 years [44,45], adults with a mean age of 39.5 ± 17 years [31], and older people with a mean age of 68.9 ± 4.28 years [40]. Four studies conducted interventions using taekwondo [40,44,45,47], one boxing [31], and one karate [46]. On the other side, two studies revealed that at baseline, their participants had no prior OCS experience [44,47], while four studies did not provide information on participants’ prior OCS experience [31,40,45,46]. 

### 3.6. Dosing and Conducted Interventions

Three studies [40,45,47] reported interventions using taekwondo of 16 weeks duration with a frequency of one to five sessions per week lasting 60 min at intensities between 50% to 80% of maximum heart rate, compared to active control groups who performed physical activity and sports [45] as physical education classes [47] and inactive control group [40]. Two studies [31,44] reported 12-week interventions with a frequency of two to four sessions per week of 50 min at intensities of 61% to 89% of maximum heart rate using boxing compared to an active control group [31] and taekwondo compared to an inactive control group [44]. Only one study [46] carried out a karate intervention lasting 36 weeks with a frequency of two sessions per week of 120 min compared to a control group that performed only physical education classes where both groups did not report the intensity of training.

In terms of activities developed in OCS interventions, four studies used taekwondo [40,44,45,47], including technical fundamentals, such as basic stances (short step, long step, and positions), displacements (forward, backward, and lateral changes), punches, blocks (low, medium, and high) and kicks (front, roundhouse kick, and descending). In one study that conducted boxing intervention by Cheema, et al. [31], a 5 min warm-up of continuous jumping was performed at a user-selected intensity. Intervals were prescribed at 2:1 (i.e., 2 min of high-intensity activity followed by 1 min of rest standing or pacing between intervals and exercises). Three intervals of each of the following five exercises were performed for 30 min of high-intensity effort: heavy bag, focus mitts, circular body bag, footwork exercises, and skipping. A study conducted by Pinto-Escalona, et al. [46] using karate intervention that began the session consisted of non-specific motor tasks aimed at improving CRF, muscle strength, coordination, balance, and flexibility. The development of the session consisted of karate-specific exercises such as kicks, bipedal and unimodal jumps, and lunges with twists. The final part of the session included stretching exercises, a discussion about the class (e.g., feelings, difficulties), and final bows.

Our systematic review found no studies that used fencing, judo, or wrestling as an intervention modality. Regarding the individuals leading the sessions with OCS, five studies reported being led by instructors or practitioners with experience in the modalities described [31,40,45,46,47], while only one did not report who led the sessions with taekwondo [44].

### 3.7. Data Collection Instruments of Cardiorespiratory Fitness (CRF)

#### 3.7.1. Direct Method

Four studies [31,44,45,47] used direct methods to measure CRF by cardiopulmonary test with spirometry. The study by Cheema, et al. [31] used a protocol starting at a predetermined for assessed adults’ comfortable walking speed for 3 min; the grade was increased by 2% every min after that until voluntary fatigue. Similarly, the study by Bae and Roh [45] used the Ebbeling protocol on a treadmill (T150; Cosmed, Rome, Italy) and wearing a wireless heart rate measuring device (Polar-a5; Polar, Kempele, Finland) in university students. Roh, Cho, and So [47] of VO_2_max were estimated with the Nemeth protocol on a treadmill (Q65, Quinton, Milwaukee, WI, USA) with wireless heart rate measuring equipment (Polar-a5, Polar, Kempele, Finland) in schoolchildren. The study by Kim, et al. [44], assessed VO_2_max with the graded exercise treadmill test with the Bruce protocol and a maximal metabolic system (Sensormedics, Yorba Linda, CA, USA) in adolescents. 

#### 3.7.2. Indirect Method

Three studies [40,44,46] used indirect methods to assess CRF. Two studies [44,46] used the 20-m shuttle run test in schoolchildren, and one study [40] used a 2-min static walking test (2MWT) in older people.

### 3.8. Outcome of Cardiorespiratory Fitness (CRF)

#### 3.8.1. Direct Method

**Figure 4:**
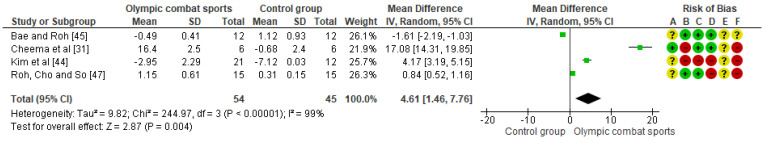
Effect of Olympic combat sports compared to control groups on the following outcome: Direct method. The squares indicate the study-specific effect estimate. Bars indicate the width of the corresponding 95%confidence interval. The diamond centered on the summary effect estimate, and the width indicate the corresponding 95% confidence interval.

Four studies [31,44,45,47] with 54 participants in OCS and 45 participants from active/inactive control were pooled, conducting a meta-analysis of VO_2_max by cardiopulmonary test with spirometry. The results indicated that OCS significantly increased VO_2_max compared to control groups (SMD = 4.61; 95% CI = 1.46 to 7.76; I^2^ = 99%; *p* = 0.004), and this is presented in Figure 4.

#### 3.8.2. Indirect Method

Meta-analyses could not be carried out due to the variability of the instruments for assessing CRF [40,44,46]. However, the study by Pinto-Escalona, et al. [46] reported significant improvements (*p* < 0.001) in the 20 m shuttle run test in favor of a 36-week karate intervention in schoolchildren compared to an active control group. On the contrary, Kim, et al. [44] did not report significant improvements (*p* ˃ 0.05) in the 20-m shuttle run test in favor of a 12-week taekwondo intervention in adolescents compared to an inactive control group. In comparison, Cho and Roh [40] reported significant improvements (*p* = 0.02) in 2MWT in favor of a 16-week intervention using taekwondo in older people compared to an inactive control group.

### 3.9. Certainty of Evidence

The certainty of evidence did not allow definitive recommendations in favor of OCS as an intervention to improve CRF in the non-athlete population (Table 4).

### 3.10. Adverse Events and Adherence

Only one study conducted by Cheema, et al. [31] reported adverse events in seven adults who participated in boxing. At the same time, five studies [40,44,45,46,47] achieved adherence equal to or greater than 70% in interventions with taekwondo and karate. One study by Kim, et al. [44] reported noncompliance with training sessions and dropout of 19 individuals due to loss of interest in the study when they were not randomly assigned to the taekwondo group.

## 4. Discussion

This systematic review aimed to analyze the effects of OCS on CRF in the non-athlete population. After reviewing 4133 records, six studies met the inclusion criteria and scored ≥ 60% in methodological quality. However, the certainty of evidence was rated as moderate to low. Therefore, it is not possible to establish a definitive recommendation for or against OCS interventions as an effective strategy for improving CRF by direct methods in the non-athlete population.

It was only possible to perform a meta-analysis on direct methods in the cardiopulmonary test with spirometry determining VO_2_max reporting statistically significant changes (SMD = 4.61; 95%CI = 1.46 to 7.76; I^2^ = 99%; *p* = 0.004) in favor of OCS groups compared to active/inactive controls. Similar to that reported by Kaya [48] in adult judo athletes, significant improvements (*p* < 0.01) in VO_2_max were reported after a strength intervention for eight weeks in anaerobic power using the Wingate test. Similarly, in a study by Kamandulis, et al. [49], inexperienced amateur boxers reported significant improvements (*p* = 0.04) in VO_2_max by spirometry in a four-week repeated sprint-specific training intervention concerning the control group performing low-intensity boxing training. Not only have these results been reported in athletes but also in the non-athlete population with pathologies as reported by Lee, Hong, and Park [42] in obese children with allergic diseases who performed a 12-week taekwondo intervention reporting significant improvements (*p* = 0.03) in VO_2_max by spirometry compared to an inactive control group. Also, Brasil, et al. [30] showed significant improvements (*p* = 0.05) in VO_2_max by spirometry in favor of a judo intervention for 12 weeks in overweight and obese schoolchildren. Other results that could not be meta-analyzed but reported individually were the indirect methods to evaluate CRF in the non-athlete population [40,44,46] showing improvements in favor of OCS in 2MWT [40] and 20-m shuttle run test [44,46]. Similar to that reported by Ojeda-Aravena, et al. [50] in young taekwondo athletes who showed significant improvements (*p* < 0.05) in CRF by 20-m shuttle run in a four-week specific in high-intensity interval training program regarding traditional taekwondo training. However, according to that reported by Herrera-Valenzuela, et al. [51] in boxer athletes, there were no significant improvements in the 1000-m test in a four-week high-intensity interval training intervention with boxing regarding a control group that only performed boxing. On the contrary, in older women, Kim, et al. [41] reported significant improvements (*p* = 0.003) in favor of a 12-week taekwondo intervention compared to an inactive control group.

A study by Hernández [52] found a significant correlation between 2MWT performance and directly measured VO_2_max in healthy adults, showing a validity of 0.92 [53]. Likewise, the 6-min walk test showed a high reliability (0.92 to 0.99) in adults with asthma [54] and healthy adults [55]. While the 20-m shuttle run test showed a high reliability of 0.98 in healthy schoolchildren and adolescents [56]. The cardiopulmonary stress test is a valid method (0.94 to 0.95) to analyze VO_2_max in older people with mild disabilities [57] and adults with lung disease [58]. While indirect calorimetry on a rolling tape is a reliable method (0.94) in apparently healthy adults [59,60], as has the ergometric tape test Bruce (0.93) in adult athletes [61], shuttle walking test (0.93) in post-stroke adults [62] and modified protocol of Balke’s rolling tape with a validity of a 0.78 in older people with chronic illnesses [63]. 

In contrast, these direct methods have proven accurate with high reliability and validity (0.78 to 0.95) in assessing CRF, such as cardiopulmonary tests with spirometry, considered the gold standard in estimating VO_2_max [64]. They are more costly and complex in their execution processes and require more time to be carried out only in laboratories, unlike indirect methods that are more economical, practical, and can be carried out in the field and also show high validity and reliability (0.93 to 0.98). Several studies [52,53,54,55,65] suggest that an alternative indirect test, such as a 6-min walk and the 2MWT and 20 m-shuttle run test, can be used as a practical and accessible alternative to estimate VO_2_max in various clinical and research settings. It should be noted that both direct and indirect CRF methods apply to individuals with different ages and characteristics.

Regarding the dosing used for OCS interventions, improvements in CRF were reported in both direct [31,44,45,47] and indirect methods [40,44,46], which reported a duration of 12 to 36 weeks with one to five weekly sessions with a time of 50 to 120 min with HR_max_ intensities of 50% to 86%. Similar to that reported in a systematic review by Franchini, et al. [66] in OCS athletes who showed significant (*p* < 0.05) increases in VO_2_max from 4.4% to 23% by ergospirometry employing an intervention of high-intensity interval training of four to seven weeks with two to five weekly sessions of 80% to 100% maximum heart rate compared to active/inactive controls. Similarly, Nam and Lim [67], in a systematic review with meta-analysis, showed a significant increase (*p* < 0.05) by 28.2% in a 200-m run test by taekwondo training regarding active/inactive controls in Korean high school athletes with an intervention dose of 12 weeks for five weekly sessions of 50 to 60 min with intensities of 50% to 80% HR_max_. These data allowed us to reinforce our findings in the present systematic review. However, it must be understood that physiological responses may vary between athletes and non-athlete populations [68]. In this systematic review, we analyzed individual reports of boxing, karate, and taekwondo in the non-athlete population; these OCS involve intermittent actions at different intensities with multidirectional movements performing strikes, grips, and turns [69], unlike athletes, with low intensities from 40% HR_max_ physiological adaptations can be generated that lead to improvements in CRF in non-athlete population performing OCS [68].

Regarding the certainty of evidence, our systematic review reported it to be moderate to low, which does not allow us to establish definitive recommendations on using OCS to improve CRF in the non-athlete population using direct methods, likewise, from that reported by Valdés-Badilla, et al. [27] in a systematic review on OCS performed by older people showing a very low-quality of evidence. Moreover, a systematic review with the meta-analysis by Ojeda-Aravena et al. [70] reported that the effects of plyometric jump training on physical fitness in combat sports athletes showed a low certainty of evidence; however, productivity in OCS had increased. For example, judo reported 383 indexed articles between 1956 to 2011 [71] and taekwondo 340 articles between 1988 to 2016 [72], while few systematic reviews have explored OCS in a non-athlete population [27], which gives strength and novelty to this systematic review.

Among the limitations of the present systematic review are (i) the diversity of instruments, values, and assessments observed in the studies, which allowed only one meta-analysis, and (ii) the diversity of the age groups analyzed. In the strengths, we found: (i) the methodological quality above 60% in the studies analyzed, (ii) the methodological processes that followed the PRISMA, PROSPERO, TESTEX, RoB, and GRADE scales, (iii) the use of seven databases: PubMed, ProQuest, EBSCOhost, CINAHL Complete, Scopus, Web of Science (core collection), PEDro, and (iv) analysis of the effect of OCS in non-athlete population. The results of this systematic review revealed that OCS interventions in non-athlete populations on CRF response are an emerging area that needs further support and research, considering that OCS is an alternative to improve health [22], showing high motivation and adherence to the practice of these sports [22,73]. Therefore, more studies with high-quality methodology (e.g., double-blind randomization, supervised control groups, and previously registered research protocols) and more description of the physical exercise (technical foundations) are needed to develop new systematic reviews, which could address other aspects of health status such as psychosociological [74], psychophysiological, physiological and/or biochemical responses to OCS interventions, and their impact on physical performance or body posture [75], as well as on central nervous system function [76] and how these effects vary with age in non-athlete population. 

## 5. Conclusions

The use of OCS improves CRF in a healthy non-athlete population of different ages, specifically showing a significant improvement in VO_2_max with direct tests, such as cardiopulmonary test. However, moderate to low certainty of evidence is reported, so definitive recommendations on using OCS interventions in the non-athlete population cannot be established. Therefore, it requires more rigorous research to comprehend the impact of OCS on the population’s health status.

## Figures and Tables

**Table 1:** Selection criteria used in the systematic review.

Category	Inclusion Criteria	Exclusion Criteria
Population	Functionally independent individuals with only one cardiometabolic risk factor (i.e., diabetes mellitus, hypertension, dyslipidemia, overweight or obesity, among others) and/or established cardiovascular or pulmonary disease.	People with sequels of cardiovascular disease of neuromuscular type (i.e., the sequel of cerebrovascular accident). Elite athletes or sportsmen.
Intervention	Interventions with OCS (boxing, fencing, judo, karate, taekwondo, wrestling) for four weeks or more.	Physical activity interventions not involving OCS.
Comparator	Interventions with a control group with or without supervised physical activity.	Lack of baseline and/or follow-up data. Absence of control group.
Outcome	At least one assessment (pre- and post-intervention) of CRF by the direct method (VO_2_max on a treadmill, ergospirometry, among others) or indirect method (6-min walk test, 2-min walk test, Chester step test, shuttle walking test, among others).	Do not present a CRF assessment.
Study design	Experimental design studies (randomized controlled trial) with pre- and post-assessment.	Non-randomized controlled trial, cross-sectional, retrospective, and prospective studies.

OCS: Olympic combat sports. CRF: cardiorespiratory fitness. VO_2_max: maximum oxygen consumption.

**Table 2:** Study quality assessment according to the TESTEX scale.

Study	Eligibility Criteria Specified	Randomly Allocated Participants	Allocation Concealed	Groups Similar at Baseline	Assessors Blinded	Outcome Measures Assessed >85% of Participants *	Intention to Treat Analysis	Reporting of between Group Statistical Comparisons	Point Measures and Measures of Variability Reported **	Activity Monitoring in Control Group	Relative Exercise Intensity Reviewed	Exercise Volume and Energy Expended	Overall TESTEX #
Cho and Roh [40]	Yes	Yes	Yes	Yes	Unclear	Yes (2)	Yes	Yes	Yes (1)	No	Yes	Yes	11/15
Cheema, et al. [31]	Yes	Yes	Yes	Yes	Yes	Yes (2)	Yes	Yes	Yes (2)	Yes	Yes	Yes	14/15
Kim, et al. [44]	Yes	Yes	No	Yes	No	Yes (1)	Yes	Yes	Yes (2)	No	Yes	Yes	10/15
Bae and Roh [45]	Yes	Yes	Unclear	Yes	Unclear	Yes (1)	Yes	Yes	Yes (2)	Yes	Yes	Yes	11/15
Roh, Cho, and So [47]	Yes	Yes	Unclear	Yes	No	Yes (1)	No	Yes	Yes (2)	No	Yes	Yes	9/15
Pinto-Escalona, et al. [46]	Yes	Yes	Yes	Unclear	Unclear	Yes (3)	Unclear	Yes	Yes (2)	Yes	Unclear	Yes	11/15

* Three points are possible: one point if adherence > 85%, one point if adverse events are reported, and one point if exercise attendance is reported. ** Two points are possible: one point if the primary outcome is reported and one point if all other outcomes are reported. # total out of 15 points. TESTEX: Tool for assessing Study quality and reporting in exercise [34].

**Table 3:** Studies report the effects of Olympic combat sports on cardiorespiratory fitness in the non-athlete population.

Study	Country	Study Design	Sample’s Initial Health	Groups	Mean Age (Year)	Type of Intervention and Control Group	Training Volume			Training Intensity	Direct Method Data Collection	Indirect Data Collection Method	Main Outcomes
				(*n*)			Weeks	Frequency (Weekly)	Time Per Session (min)				
Cho and Roh [40]	South Korea	RCT	Older people apparently healthy	EG: 19 CG: 18	EG: 68.89 ± 4.16 CG: 69.00 ± 4.41	EG: Taekwondo CG: usual activities	16	5	60	50–80% HR_max_	NR	2MWT	EG vs. CG ↑ 2MWT in favor of EG
Cheema et al. [31]	Australia	RCT	Adults apparently healthy	EG: 6 CG: 6	EG: 43 ± 19 CG: 36 ± 15	EG: Boxing CG: Walking	12	4	50	EG: 86–89% HR_max_ CG: 64–77% HR_max_	Indirect calorimetry using a standard ramp protocol on a laboratory treadmill	NR	EG vs. CG ↑ VO_2_max in favor of EG
Kim et al. [44]	United States of America	RCT	High school females apparently healthy	EG: 21 CG: 10	EG: 15.7 ± 0.4 CG: 15.9 ± 0.6	EG: Taekwondo CG: usual activities	12	2	50	61% HR_max_	Ergometric tape test Bruce.	20-m shuttle run test	EG vs. CG ↔ VO_2_max EG vs. CG ↑ 20-m shuttle run test in favor of EG
Bae and Roh [45]	South Korea	RCT	University students apparently healthy	EG: 12 CG: 12	EG: 22.42 ± 4.40 CG: 23.25 ± 4.31	EG: Taekwondo CG: Physical activity and recreational sports	16	1	60	50–80% HR_max_	CP test spirometry (Ebbeling protocol)	NR	EG vs. CG ↔ VO_2_max
Pinto-Escalona et al. [46]	Multicenter (Spain, Portugal, France, Poland and Germany)	RCT	Schoolchildren apparently healthy	EG: 388 CG: 333	EG: 7.4 ± 0.5 CG: 7.4 ± 0.4	EG: Karate CG: Physical education classes	36	2	120	NR	NR	20-m shuttle run test	EG vs. CG ↑ 20-m shuttle run test in favor of EG
Roh, Cho, and So [47]	South Korea	RCT	Schoolchildren apparently healthy	EG: 15 CG: 15	EG: 11.53 ± 0.64 CG: 11.40 ± 0.63	EG: Taekwondo CG: Physical education classes	16	1	60	50–80% HR_max_	CP test with spirometry (protocol of Nemeth)	NR	EG vs. CG ↔ VO_2_max

RCT: randomized controlled trial. NR: not reported. *n*: number. EG: experimental group. CG: control group. HR_max_: maximum heart rate. 2MWT: 2-min walk test: 6MWT: 6-min walk test. CP: cardiopulmonary. CPET: cardiopulmonary stress test. VO_2_max: maximum oxygen consumption. ↑: significant improvement. ↔: no significant difference.

**Table 4:** GRADE assessment for the certainty of evidence.

Outcome	Study Design	Risk of Bias in Individual Studies	Risk of Publication Bias	Inconsistency	Indirectness	Imprecision	Certainty of Evidence	Recommendation
Direct method	4 RCT and 97 participants	Moderate to high ^1^	High ^3^	Moderate ^4^	Moderate ^5^	Moderate to high ^6^	Moderate to low ^8^	The available evidence did not allow definitive recommendations on using OCS to improve CRF in the non-athlete population.
Indirect method	2 RCT and 758 participants	High ^2^	High ^3^	Moderate ^4^	Moderate ^5^	High ^7^	Low ^9^	

^1^ Three studies showed a high risk of individual bias, and only one showed some concerns. ^2^ All studies showed a high risk. ^3^ All studies show a high risk of publication. ^4^ High statistical heterogeneity (assessed through I^2^) and/or high clinical or methodological heterogeneity (interventions and study designs). ^5^ In our study, measurements were performed directly, so no surrogate results were used. The population (non-athletes, apparently healthy) was clearly defined and corresponded to our objectives. ^6^ Very large 95% confidence intervals and only one study performed power analysis and sample calculation. ^7^ Very large 95% confidence intervals. ^8^ Moderate to high (risk of bias in individual studies), high (risk of publication bias), moderate (inconsistency, indirectness), and moderate to high (imprecision). ^9^ High (risk of bias in individual studies and risk of publication bias); Moderate (inconsistency and indirectness) and high (imprecision). OCS: Olympic combat sports. CRF: cardiorespiratory fitness.

## Data Availability

The data set generated and/or analyzed during the current revision is available for any reasonable request.
